# The Impact of the *Tips from Former Smokers®* Campaign on Reducing Cigarette Smoking Relapse

**DOI:** 10.1155/2022/3435462

**Published:** 2022-11-22

**Authors:** Kevin Davis, Rebecca Murphy-Hoefer, Lauren Dutra, Brian King, Brian Bradfield, Robert Rodes, Diane Beistle

**Affiliations:** ^1^Center for Health Analytics, Media, And Policy, RTI International, 3040 E Cornwallis Rd, Durham, NC 27709, USA; ^2^Office of Smoking and Health, Centers for Disease Control and Prevention, 1600 Clifton Rd, Atlanta, GA 30333, USA

## Abstract

Evidence-based mass-reach health communication campaigns can increase tobacco cessation, use of cessation resources such as quitlines, and change tobacco-related social norms. These interventions have been associated with a lower likelihood of cigarette smoking relapse in studies conducted internationally; however, no studies have assessed this outcome for a national campaign in the United States. This study examined the relationship between *Tips from Former Smokers®* (*Tips®*) campaign exposure and the odds of cigarette smoking relapse among adults who formerly smoked. Using data from the 2014 to 2019 *Tips* longitudinal campaign surveys, we estimated first episode of relapse (versus remaining a former smoker) as a function of *Tips* gross rating points (GRPs, a measure of media exposure). Higher levels of *Tips* GRPs were associated with lower odds of relapse (aOR = 0.63, 95% CI: 0.50-0.78). These results suggest that the *Tips* campaign may reduce smoking relapse, in addition to the established effect of increasing smoking cessation. Former smokers can be considered a secondary target audience for smoking cessation mass media campaigns, and mass media campaigns could be considered a component of smoking relapse prevention efforts.

## 1. Introduction

Cigarette smoking remains the leading cause of preventable disease, disability, and death in the United States [1]. According to the 2020 Surgeon General's Report, smoking cessation reduces the risk of premature death and can add as much as a decade to life expectancy [1]. More than two-thirds of U.S. adults who smoke cigarettes report interest in quitting cigarette smoking [2]. Sustained efforts to increase access to and use of evidence-based cessation treatments among adults who smoke, in coordination with population-based interventions, are important to effectively address the continuum of tobacco use including initiation, cessation, and relapse [1].

Population-based interventions to address tobacco use include evidence-based mass-reach health communication campaigns, which have been shown to increase cigarette smoking cessation, use of cessation resources such as quitlines, and change tobacco-related social norms [3–5]. The Centers for Disease Control and Prevention's (CDC's) national *Tips from Former Smokers® (Tips®)* campaign is the first federally funded national mass media campaign in the U.S. to encourage adults who smoke to quit and make free help available. *Tips®* shares real life stories of people who are living with serious long-term health effects due to smoking cigarettes and secondhand smoke exposure on a variety of media channels including television, radio, print, and digital media [6]. *Tips®* has been associated with quit attempts and smoking cessation among adults who currently smoke [7–10]. From 2012 to 2018, the *Tips*® campaign was associated with an estimated 16.4 million quit attempts and over one million sustained quits among adults who smoked cigarettes [9].

Although extensive research has been conducted on relationship between *Tips*® campaign exposure and cigarette smoking cessation among current smokers, no study has examined its potential preventative effects on cigarette relapse among former smokers. Existing studies from Australia [11] and England [12] have reported that, in addition to helping people quit smoking, exposure to mass media campaigns has been associated with a lower likelihood of relapse among people who had recently quit smoking. However, no study has assessed the impact of a national campaign on reducing relapse in the United States. To address this gap, this study examined the relationship between *Tips* campaign exposure and the odds of relapse to cigarette smoking among people who formerly smoked.

## 2. Materials and Methods

### 2.1. Data and Sample

This study's sample and recruitment methods are based on those of previous studies examining the effectiveness of the *Tips* campaign [5, 8, 10, 13]. In brief, we used data from a longitudinal online panel of adult cigarette smokers in the U.S. Adult cigarette smokers were identified as individuals who reported currently smoking cigarettes either “some days” or “every day” at the time of initial recruitment. Respondents were recruited from a combination of the nationally representative Ipsos KnowledgePanel® (KP) and a custom Ipsos panel recruited using the same methods as KP. Both samples are drawn from an address-based random sample (ABS) of U.S. households covering approximately 95% of the U.S. adult population (18 years and older). Respondents received invitation packets containing study information and unique links to join the study. Respondents to the initial survey (Spring 2014) were followed longitudinally and invited to participate in subsequent surveys, with new sample periodically recruited via ABS to replenish respondents lost to follow-up. The combined sample was weighted to reflect age, sex, race/ethnicity, and educational distributions among adult cigarette smokers from the National Health Interview Survey.

Data collection was timed to coincide with the *Tips* campaign each year. Approximately three surveys were conducted per year in 2014, 2015, 2016, and 2018, with one survey wave being conducted in 2019. No data were collected in 2017. Survey waves were conducted approximately 3-6 months apart and, each year, were timed to occur before campaign launches, during the campaigns, and after the campaigns ended. In total, 12 waves of survey data were collected between 2014 and 2019.

Because this study examines cigarette relapse as the primary outcome, we further limited the cigarette smoker panel data to the subset of respondents who transitioned to nonsmoker status during the study period (i.e., recent former smokers). Recent former smokers were defined as current cigarette smokers who reported smoking “not at all” in at least one subsequent survey over the course of their tenure in the panel. This subset of recent former smokers was then further limited to respondents who recorded their cigarette smoking status for all remaining survey waves after first reporting former smoking. We excluded participants from the analytic sample who missed all survey waves after transitioning to former smoking, since we could not determine whether they relapsed after the transition to former smoking (20.1% of the initial former smoking sample). All formerly smoking respondents in the analytic sample completed a minimum of 3 survey waves and up to a maximum of 12, averaging approximately 9 completed waves per respondent (SD = 2.1). Including all repeated measurements on individual participants, the final analytic data consisted of 3,464 total observations on 1,409 unique former smokers.

### 2.2. Outcome Variable: Cigarette Smoking Relapse

The outcome variable was cigarette relapse, measured for the analytic sample of recent former smokers. Respondents who remained former smokers at their first follow-up survey were assigned a value of relapse = 0 for that wave and all applicable waves thereafter. Former smokers who indicated relapsing to current cigarette smoking at a given wave were assigned a value of relapse = 1 for that wave and then given missing values for relapse for all waves thereafter. Former smokers who continued to report former smoker status in all remaining follow-up survey waves were assigned a value of relapse = 0.

Assignment of missing values for all survey waves after cigarette relapse simulates survival analysis by censoring the outcome [14]. That is, the analytic sample is restricted to only those participants who are “at risk” of cigarette relapse, while still applying logistic regression to the dataset [15]. This approach allows us to present odd ratios for campaign effects on cigarette relapse, which are more easily interpreted than the hazard ratios generated by standard survival analyses.

### 2.3. Exposure Variable: *Tips* Campaign Gross Rating Points

Respondent exposure to the *Tips* campaign was measured as past-quarter cumulative *Tips* Gross Rating Points (GRPs), a measure of media campaign dose that varies by respondents' designated market area (DMA) and time. Past-quarter GRPs were merged to the survey data based on respondents' DMA of residence and survey completion date. Following the methods used in previous studies, GRPs were transformed into curvilinear form (by taking the square root) to capture expected diminishing marginal effects of GRPs over the range of observed GRPs [5].

### 2.4. Covariates

Covariates included in the analysis were as follows: sex (male or female); age in years; race/ethnicity (non-Hispanic white, non-Hispanic Black, Hispanic, or non-Hispanic other race/multiracial); education (less than high school, high school degree or General Education Diploma (GED), some college but no degree or an associate's degree, and a college education (a Bachelor's degree or greater); most or all of the respondent's family members or friends smoke (versus most of them are not smoking or none of them are smoking); annual household income (in tens of thousands of U.S. dollars); living with one or more persons who smoke cigarettes; having one or more children in the household; having one or more physical health conditions (endorsing being diagnosed with one or more of 19 listed medical conditions, which ranged from acid reflux disease to stroke); and having one or more mental health conditions (reporting prior diagnosis of ADHD or ADD, anxiety disorder, depression, and/or “mental health condition”). Covariates were measured at each survey wave, allowing for covariate variation over time. The covariates we chose are based on those used in a broader literature that examines the impact of the *Tips* campaign and has found many of these covariates to be significantly associated with cigarette cessation-related outcomes [5, 8, 9, 10, 13, 16]. For regression models, we included all covariates that significantly improved model fit (as measured by *R*^2^). Any missing values on covariates at a given wave were replaced with values from the most recent wave they were measured for each individual respondent. Lastly, we created separate indicator variables for each state to account for time-invariant state characteristics (i.e., state fixed effects).

### 2.5. Statistical Analysis

We used descriptive statistics to characterize the sample at the first wave of data included in this analysis. Logistic regression was used to model relapse as a function of *Tips* GRPs. To adjust the standard errors for clustered values within each individual, we used Stata's (Stata Version 17) “svy” survey data suite of commands to specify the unique respondent ID as the clustered ID variable for the logistic regression. In addition, we applied survey weights, also using the “svyset” statement in Stata and then performed the logistic regression using the “svy: logistic” command, which generates weighted, adjusted odds ratios for GRPs that account for correlated data within unique individual respondents.

GRPs were scaled so that odds ratios reflected the difference in odds of relapse for a 1,000 quarterly GRP increase in *Tips* exposure. This GRP increment was chosen because it reflects the typical quarterly *Tips* campaign ad buy and the CDC's best practice recommendations for effective tobacco prevention campaigns [3]. All models were weighted and included all covariates described previously.

To visually illustrate the relationship between GRPs and relapse, we also used the regression model results to calculate the predicted probability of relapse associated with past-quarter GRPs, averaged across markets and time, for all GRP values observed during the study period (0 to 4,072). Because no surveys were conducted in 2017, we conducted a sensitivity analysis to determine the potential impact of missingness on our results. To do this, we reran the regression analysis on 2014-2016 data alone, excluding the 2018 and 2019 survey waves, to assess whether the pre-2017 data yielded different results from the complete 2014–2019 data. This analysis helped establish whether the lack of survey data in 2017 introduced significant bias in analysis of the overall 2014–2019 time period.

## 3. Results

### 3.1. Sample Characteristics

Weighted sample characteristics are summarized in Table 1. The sample was 53.5% male and 46.5% female with a mean overall age of 43.8 years. Participants who reported being non-Hispanic white comprised 64.4% of the sample, and participants who reported being non-Hispanic Black comprised 14.5% of the weighted sample. Participants who reported being Hispanic and non-Hispanic other race/multiracial participants made up the remaining 15.2% and 6.0% of the sample, respectively. Respondents with less than a high school degree comprised 13.0% of the sample, and those with a high school degree or a GED comprised 42.5% of the sample. Participants with some college education comprised 31.1% of respondents and those with a bachelor's degree or higher comprised the remaining 13.4% of the sample. Approximately 54.5% of respondents reported that most or all of their family or friends smoked.

Approximately half (50.5%) of the analytic sample reported relapsing to current smoking during the follow-up period. Once participants transitioned to former smoking, for those who reported relapse, the average time to reporting first relapse was approximately 9.5 months (mean = 288.69 days, SD = 281.73). Among respondents reporting former smoking who did not report relapse during the follow-up period, the average length of time spent as a former smoker before the end of data collection (2019) was approximately 2.6 years (mean = 958.42 days, SD = 495.16). Past-quarter cumulative *Tips* GRPs averaged 772 and ranged from 0 to 4,072 among survey respondents across the study timeframe.

### 3.2. Association between *Tips* GRPs and Cigarette Relapse

Adjusting for covariates, higher levels of *Tips* GRPs were associated with lower odds of cigarette relapse (adjusted odds ratio (aOR) = 0.63, 95% CI: 0.50-0.78; Table 2). Specifically, each 1,000 GRP increase was associated with a 37% decrease in the odds of relapse. Other significant covariates included reporting that most or all family or friends smoke, which was associated with higher odds of relapse (aOR = 2.40, 95% CI: 1.75-3.30). Except for several state fixed effects, the remaining covariates in the model were not significant.

**Figure 1:**
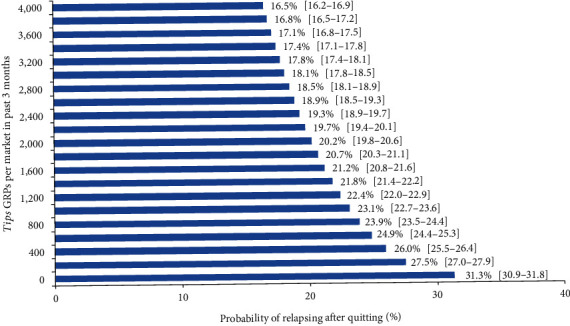
Model-predicted^a^ cigarette smoking relapse by mean observed past-quarter *Tips* campaign GRPs (95% CI) among former smoking^b^ adults. ^a^The analytic model adjusted for weights and the following covariates: sex, race/ethnicity, education, having family or friends who smoke, household income, whether one or more smokers live in the household, whether one or more children live in the household, whether participants have physical health conditions, and whether participants have mental health conditions. ^b^Former smokers were participants who reported smoking “every day” or “some days” (current smoking) in Spring 2014 and subsequently reported smoking “not at all” for at least one wave thereafter.

Figure 1 illustrates the dose-response relationship between GRPs and relapse using the model-predicted probabilities of relapse across the range of mean past-quarter *Tips* GRPs (averaged across markets and time) observed during the study period. The predicted probabilities of relapse ranged from 16.5% (95% CI: 16.2%-16.9%) at 4,000 GRPs to 31.3% (95% CI: 30.9%-31.8%) at 0 *Tips* GRPs (i.e., no campaign). The predicted probability of relapse at an average *Tips* dose of 1,000 quarterly GRPs was 23.1% (95% CI: 22.7%-23.6%).

### 3.3. Sensitivity Analysis Results

Restricting the dataset to pre-2017 data reduced the model sample size from 3,464 to 2,776 observations. The covariate-adjusted odds of relapse resembled the full model results (aOR = 0.62, 95% CI: 0.48-0.80). The directionality and significance of all covariates remained the same.

## 4. Discussion

Our study found that higher levels of *Tips* GRPs were associated with lower odds of relapse to cigarette smoking among U.S. adults who formerly smoked cigarettes. Although the *Tips* campaign primarily is aimed at increasing quit attempts among individuals who currently smoke, the results of our analysis suggest *Tips* may be protective against cigarette smoking relapse among those who have quit. In addition to existing evidence documenting the impact of mass-reach health communication campaigns on smoking cessation in the U.S., these findings indicate an additional function of the campaign in potentially reducing cigarette smoking relapse.

In addition to our U.S.-focused analysis, several other studies have examined the impact of mass media campaigns on maintaining abstinence from cigarette smoking or preventing relapse [11, 12, 17]. Biener et al. [17] found that, in Massachusetts, among individuals who reported quitting smoking in the past 2 years, participants who reported remaining abstinent for more than 6 months were more likely to report that TV advertisements had helped them quit smoking compared to participants who reported remaining abstinent for 6 months or less. Consistent with our findings, a national prospective study in Australia found that greater exposure to mass media campaigns was associated with lower odds of relapse among those who recently quit and that “keeping the reasons for quitting salient can help ex-smokers resist temptations to smoke, resist urges to smoke, and reinforce the value of quitting” [11]. Similarly, in England, researchers found that, among adults who reported former smoking, a significantly greater percentage of adults exposed to a national antismoking TV campaign (featuring tips on preventing relapse) remained abstinent at 18 months follow-up compared to those not exposed to the campaign [12]. Secondary outcome studies in New York [18] and Florida [19] suggested that exposure to cessation media messages may also influence smoking relapse, but these studies did not find a statistically significant relationship between campaign exposure and relapse. Possible explanations for the difference between our findings and theirs are the lack of measurement of advertising exposure between baseline and follow-up in the New York study [11, 18] and the lower than recommended campaign target rating points in the Florida study [19].

One possible explanation for our significant findings on cigarette smoking relapse is *Tips*' use of emotive messaging focused on the negative health consequences of cigarette smoking [11, 12, 17, 20, 21]. Other campaign ads with similar themes as *Tips* have been found to reduce smoking relapse [9, 11, 22]. In Massachusetts, researchers found that ads focused on the health consequences of cigarette smoking or “inspirational quit tips” were most helpful for preventing relapse [17]. Similarly, in Australia, ads focused on the negative health consequences of smoking cigarettes helped persons who recently quit to maintain their nonsmoking status [11].

In the context of existing studies, our findings have important implications for considering individuals who formerly smoked as part of the core target audience, in addition to those who currently smoke, when developing messages for tobacco control mass media campaigns. Most people who attempt to quit smoking relapse [2] take as many as 30 attempts to quit successfully [1]. Accordingly, cessation maintenance has been identified as an important step in staying quit [23]. Our study also draws attention to the benefit of combining individual-level interventions, which are well-documented [1, 24], with mass media campaigns as part of relapse prevention initiatives. Our findings support existing evidence for a comprehensive approach that uses effective message themes and executional styles, campaigns with sufficient reach and duration, and maintaining social norm influences to reduce smoking relapse [3, 25]. These interventions have the potential to support smoking abstinence for the estimated 55 million adults in the U.S. who have quit smoking [26].

In addition, our findings add to an already substantial body of evidence that the *Tips* campaign is effective, including its association with over one million sustained quits [9], economic savings and gains in life quality [27, 28], increased calls to quitlines and visits to cessation-related websites [21, 29], and knowledge of tobacco-related health risks [30, 31]. Previous research has focused on the economic savings associated with smoking cessation that has been attributed to *Tips* [27, 28]. Our findings suggest that a reduction in relapse may be associated with *Tips*. Future research could quantify the health-related economic benefits associated with this reduction in relapse to current smoking attributable to *Tips*.

### 4.1. Limitations

This analysis has several limitations that should be considered. The primary limitation is that there were no surveys collected in 2017. Because of this gap in the data, participants who relapsed to current cigarette smoking in 2017, then returned to former smoking during the 2018 evaluation, could be misclassified as formerly smoking in our analysis. However, our sensitivity analysis that reexamined the relapse model using pre-2017 data produced similar results to the overall model. This finding suggests that the 2017 data gap was unlikely to be a major source of bias resulting from misclassifications of relapse patterns. Another limitation of this analysis is that we did not capture additional quit attempts after the first relapse; it is likely that some of the respondents who relapsed to smoking during the study period later quit. In addition, the analysis did not include information about respondent behavior after the end of the study period; as a result, we were not able to capture smoking relapses that occurred after the final survey wave. Another limitation is our use of self-reported data, which can be prone to misreporting of smoking status and relapses that have occurred. However, the short time periods between waves of data collection for *Tips* (except for 2017) and the literature establishing self-reported smoking status as a reliable measure [32] make the possibility of uncaptured relapses less likely. Lastly, this analysis only accounts for *Tips* GRPs, which capture *Tips* exposure via TV, and does not include other possible sources of exposure to *Tips*, such as digital media; however, TV is the largest component of the campaign's media buy and thus the primary source of *Tips* exposure.

## 5. Conclusions

Higher levels of exposure to the *Tips* campaign were associated with lower odds of relapse to cigarette smoking. The finding of a dose-response relationship between *Tips* exposure and relapse supports the role of tobacco cessation mass media campaigns in reducing the risk of cigarette smoking relapse and identifies an additional beneficial outcome of the *Tips* campaign. Evidence-based mass-reach health communication campaigns that promote cessation and prevent relapse are an important part of a comprehensive approach to increase and maintain cigarette smoking cessation.

## Figures and Tables

**Table 1:** Spring 2014 unweighted and weighted^a^ characteristics of participants in the analytic sample^b^.

Characteristic	*n* ^c^	Unweighted statistics *n* (%)/mean (SD)	Weighted statistics % (95% CI)/mean (SE)
Past-quarter cumulative TV GRPs	1,409	756.32 (346.25)	771.98 (14.73)
Sex	1,409		
Female		784 (55.6%)	46.5% (42.5%-50.5%)
Male		625 (44.4%)	53.5% (49.5%-57.5%)
Age	1,409	50.77 (15.32)	43.76 (0.61)
Race/ethnicity	1,409		
Non-Hispanic white		1,134 (80.5%)	64.4% (60.0%-68.7%)
Non-Hispanic black		107 (7.6%)	14.5% (11.1%-17.8%)
Hispanic		95 (6.7%)	15.2% (11.4%-18.9%)
Non-Hispanic other race/multiracial		73 (5.2%)	6.0% (3.8%-8.2%)
Education	1,407		
Less than high school		91 (6.5%)	13.0% (9.8%-16.3%)
High school graduate or GED		347 (24.7%)	42.5% (38.3%-46.7%)
Some college (an associate degree or no degree)		636 (45.2%)	31.1% (27.8%-34.4%)
Bachelor's degree or higher		333 (23.7%)	13.4% (11.1%-15.7%)
Other characteristics			
Most or all family or friends smoke	1,409	683 (48.5%)	54.0% (50.0%-58.1%)
Household income ($)	1,365	53,700 (37,900)	54,100 (0.16)
≥1 smokers in household	1,402	444 (31.7%)	33.4% 29.7%-37.1%)
≥1 child in household	1,409	382 (27.1%)	38.5% (34.4%-42.7%)
≥1 physical health conditions	1,408	1,056 (75.0%)	64.5% (60.4%-68.6%)
≥1 mental health conditions	1,401	423 (30.2%)	27.9% (24.3%-31.5%)

Abbreviations: TV: television; GRPs: gross rating points; SD: standard deviation; CI: confidence interval; SE: standard error; GED: general education degree. ^a^Weighted to reflect age, sex, race/ethnicity, and education benchmark distributions among adult cigarette smokers from the National Health Interview Survey. ^b^To be included in the analytic sample, participants had to report smoking “every day” or “some days” (current smoking) in the initial survey wave, subsequently report smoking “not at all” for at least one wave thereafter. ^c^The sample size represents the number of unique participants with nonmissing values at the initial survey wave.

**Table 2:** Adjusted odds of relapsing to current cigarette smoking among former smokers^a^ (model *n* = 3,464, unique participants = 1,409)^b^.

Model covariate	aOR (95% CI)	**p** value	
Curvilinear past-quarter tips GRPs	0.63 (0.50-0.78)	<0.001	
Sex			
Female	1 (reference)		
Male	1.01 (0.74-1.37)	0.948	
Age	1.00 (0.99-1.01)	0.445	
Race/ethnicity			
Non-Hispanic white	1 (reference)		
Non-Hispanic black	0.87 (0.53-1.43)	0.579	
Hispanic	1.23 (0.70-2.15)	0.469	
Non-Hispanic other race/multiracial	1.12 (0.59-2.13)	0.726	
Education			
Less than high school	1 (reference)		
High school degree or GED	0.73 (0.40-1.32)	0.294	
Some college (an associate degree or no degree)	0.71 (0.40-1.26)	0.237	
Bachelor's degree or higher	0.68 (0.36-1.28)	0.234	
Other characteristics			
Most or all family or friends smoke	2.40 (1.75-3.30)	<0.001	
Household income	1.00 (0.96-1.05)	0.862	
≥1 smokers in household	1.14 (0.82-1.59)	0.433	
≥1 child in household	1.23 (0.87-1.73)	0.236	
≥1 physical health conditions	1.19 (0.82-1.71)	0.360	
≥1 mental health conditions	1.02 (0.75-1.40)	0.882	

Abbreviations: aOR: adjusted odds ratio; CI: confidence interval; GRPs: gross rating points; GED: general education degree. ^a^Former smokers were participants who reported smoking “every day” or “some days” (current smoking) in Spring 2014 and subsequently reported smoking “not at all” for at least one wave thereafter. ^b^The analysis was weighted to reflect age, sex, race/ethnicity, and education benchmark distributions among adult cigarette smokers from the National Health Interview Survey.

## Data Availability

Readers can access the data supporting the conclusions of the study by sending an inquiry to tobaccomediacampaign@cdc.gov.
